# ANXA1 enhances tumor proliferation and migration by regulating epithelial-mesenchymal transition and IL-6/JAK2/STAT3 pathway in papillary thyroid carcinoma

**DOI:** 10.7150/jca.52171

**Published:** 2021-01-01

**Authors:** Xin Zhao, Weiguo Ma, Xinyu Li, Haijun Li, Jin Li, Hongle Li, Fucheng He

**Affiliations:** 1 Department of Medical Laboratory, The First Affiliated Hospital of Zhengzhou University, Zhengzhou, Henan 450000, China; 2 Department of Medical Laboratory, The Affiliated Cancer Hospital of Zhengzhou University, Zhengzhou, Henan 450008, China; 3 Department of Molecular Pathology, The Affiliated Cancer Hospital of Zhengzhou University, Zhengzhou, Henan 450008, China.

**Keywords:** ANXA1, papillary thyroid cancer, epithelial-mesenchymal transition, IL-6, STAT3

## Abstract

**Background:** Annexin A1 (ANXA1) was discovered to show various effects during tumor initiation and development in a tumor-specific manner. However, the function of ANXA1 in papillary thyroid carcinoma (PTC) has not been reported.

**Methods:** Bioinformatic analyses, RT-PCR and immunohistochemistry were employed to determine the ANXA1 expression level in PTC. Both gain- and loss-of-function studies, including CCK-8, EdU assay, transwell experiment and wound-healing assay were used to investigate the role of ANXA1 in PTC progression. GSEA enrichment analysis was utilized to explore the potential mechanisms of ANXA1 mediated downstream signaling, and ELISA, RT-PCR and western blot were used to confirm the relevance.

**Results:** ANXA1 expression was prominently upregulated in PTC tumor tissues. Ectopic expression of ANXA1 expedited PTC cell proliferation, migration and invasion, whereas ANXA1 knockdown exhibited the opposing trends. Mechanistic investigations showed that ANXA1 regulated epithelial-mesenchymal transition (EMT) and activated the IL-6/JAK2/STAT3 pathway to contribute to PTC malignant behaviors. In particular, loss of ANXA1 retarded tumor burden and suppressed lung metastasis *in vivo*.

**Conclusions:** In conclusion, our findings identified ANXA1 as a pivotal oncogene during PTC carcinogenesis and ANXA1 might function as a promising therapeutic target and prognostic marker for PTC.

## Introduction

Thyroid cancer (TC) represents the most prevalent endocrine tumor. It is the ninth most common malignancy and one of the most rapidly increasing cancers around the world 1. In general, up to 80-85% of thyroid cancers are well-differentiated papillary thyroid carcinoma (PTC), with a 10-year survival rate of above 90% 2. However, a small fraction of PTCs dedifferentiate into the aggressive type, characterized by multiple metastases at early stages and cannot be entirely cured by current treatment options such as surgery and radioiodine. Moreover, 10% of PTC patients experienced recurrence, and 5-10% of patients may suffer from distant metastasis 3, 4. To this end, it is urgent to identify novel treatment targets for PTC and understand the molecular mechanisms during PTC initiation and progression.

Annexin A1 (ANXA1) belongs to the annexin superfamily, functioning as a calcium-dependent phospholipid-binding protein and building a bridge between Ca^2+^ signaling and membrane functions 5. The annexin superfamily contains 13 members with high biological and structural homology owing to their similar C-terminal domain 6. ANXA1 was the first identified member of this family and found to participate in the anti-inflammatory effect of glucocorticoids through suppressing the activity of cytosolic phospholipase A2 (cPLA2) and cyclooxygenase 2 (COX-2) 7. Besides, exogenous ANXA1 accelerated polymorphonuclear neutrophils (PMN) apoptosis by provoking intracellular calcium concentrations and inducing dephosphorylation of BAD, thereby allowing BAD to associate with the mitochondria 8. With continuous more in-depth research in this field, ANXA1 has been revealed to play regulatory roles in a variety of cellular processes during tumor progression. The first study linking ANXA1 to cancer was documented in lung cancer, in which ANXA1 exhibited inhibitory effects on A549 cell growth and leukocyte migration 9. Differential expression of ANXA1 was found in multiple cancers. It was reported to be elevated in hepatocellular carcinoma, breast cancer, gastric cancer and melanoma, while decreased in nasopharyngeal carcinoma, prostate cancer and esophageal carcinoma, indicating the context-dependent role for ANXA1 in carcinogenesis 10-15. Petrella et al. previously found that the expression of ANXA1 was higher in PTC and follicular thyroid cancer (FTC) cell lines and specimens compared to undifferentiated thyroid cancer (UTC) samples and cell lines, suggesting ANXA1 may represent a useful differentiation marker in thyroid cancer 16. However, the specific function of ANXA1 in PTC remains mostly elusive.

In the present study, we elucidated that ANXA1 was markedly increased in PTC compared with normal thyroid tissues, whose expression is closely related to the larger tumor size and poor survival of PTC patients. Overexpression of ANXA1 aggravated the proliferation and metastasis of PTC cells both *in vitro* and *in vivo*. Mechanistic studies revealed that ANXA1 regulated PTC malignant phenotypes through epithelial-mesenchymal transition (EMT) and IL-6/JAK2/STAT3 pathway. In summary, our findings delineated the biological function, clinical significance and molecular mechanisms of ANXA1 in PTC progression.

## Materials and Methods

### Human Samples and cell lines

PTC specimens and corresponding normal thyroid tissues from 64 patients were collected from the First Affiliated Hospital of Zhengzhou University. All enrolled patients were confirmed without therapeutic intervention such as chemo- or radioiodine therapy before surgery and were provided with written informed consent. The pathological examination of tumor tissues was confirmed by two independent pathologists and classified according to the TNM staging system. Normal thyroid epithelial cell line Nthy-ori 3-1 and human PTC cell lines BCPAP, TPC-1 and KTC-1 were purchased from Chinese Academy of Sciences and preserved in a 37 °C humidified incubator with 5% CO_2_. All cells were cultured in Dulbecco's Modified Eagle's Medium (DMEM, Biological Industries, USA) supplemented with 1% non-essential amino acid (NEAA, Gibco, USA) and 10% fetal bovine serum (FBS, Biological Industries, USA) and cryopreserved in CELLSAVING (New Cell & Molecular Biotech, Suzhou, China) at -80 °C. Cell culture plates and dishes were purchased from Hangzhou Xinyou Biotechnology Co., Ltd.

### RT-PCR

Total RNAs from PTC tissues and cells were isolated using RNeasy Mini kit (Qiagen, USA) and 1 μg of RNA was used for reverse transcription with PrimeScript RT reagent Kit with gDNA Eraser (TaKaRa, Japan). Real-time quantitative PCR was performed with Power SYBR green PCR master mix (Applied Biosystems) and mRNA expression was normalized by the expression of β-actin and analyzed based on the relative quantification method 2^-ΔΔCt^. The primers for each gene were listed as follows: ANXA1 forward: GCGGTGAGCCCCTATCCTA, reverse: TGATGGTTGCTTCATCCACAC. E-cadherin forward: CGAGAGCTACACGTTCACGG, reverse: GGGTGTCGAGGGAAAAATAGG. ZO-1 forward: CAACATACAGTGACGCTTCACA, reverse: CACTATTGACGTTTCCCCACTC. N-cadherin forward: AGCCAACCTTAACTGAGGAGT, reverse: GGCAAGTTGATTGGAGGGATG. Vimentin forward: AGTCCACTGAGTACCGGAGAC, reverse: CATTTCACGCATCTGGCGTTC. IL-6 forward: ACTCACCTCTTCAGAACGAATTG, reverse: CCATCTTTGGAAGGTTCAGGTTG. β-actin forward: CATGTACGTTGCTATCCAGGC, reverse: CTCCTTAATGTCACGCACGAT.

### Plasmids construction

The cDNA of ANXA1 was amplified and introduced into the pCDH-CMV plasmid, while the empty pCDH-CMV vector was used as control. For inhibition of ANXA1, shRNA sequences targeting ANXA1 were inserted in pLKO.1-puro vector and a scramble shRNA was utilized as control. These lentiviral plasmids were co-transfected with packaging vectors into 293T cells using Lipofectamine 3000 (Invitrogen, USA). Two days after transfection, virus-containing medium was collected to infect target PTC cell lines. Puromycin (4 μg/mL) was added to select stable ANXA1 knockdown or overexpression cells for one week.

### Immunoblotting

PTC cells were lysed using the RIPA buffer with phosphatase inhibitor cocktail on ice for 30 minutes and centrifuged for 15 minutes at 4 °C, and the supernatants were collected for quantification using BCA Protein Assay Kit (Thermo, USA). The following antibodies were used to measure protein levels in this study: anti-ANXA1 (ab33061, Abcam), anti-E-cadherin (#3195, Cell Signaling Technology), anti-ZO-1 (#8193, Cell Signaling Technology), anti-N-cadherin (#13116, Cell Signaling Technology), anti-Vimentin (#5741, Cell Signaling Technology), anti-JAK2 (#3230, Cell Signaling Technology), anti-p-JAK2 (Tyr1007/1008, #3771, Cell Signaling Technology), anti-STAT3 (#12640, Cell Signaling Technology), anti-p-STAT3 (Tyr705, #9145, Cell Signaling Technology) and anti-GAPDH (bs-2188R, BIOSS).

### Cell viability assays

Cell viability was conducted with cell counting kit-8 (CCK-8, Dojindo, Japan) assay. A total of 2000 cells were plated into each well on 96-well culture plates. 10 µL CCK-8 solution was added to 90 μL of the DMEM medium in each well every day and the absorbance was measured at 450 nm on a microplate reader. Cell growth was also investigated by EdU assay kit (RiboBio, China). 5000 PTC cells with indicated vectors were seeded into 96-well plates. After cell attachment, 50 µM EdU reagent was added into each well and incubated at 37 ˚C for 3 hours. Edu positive cells were visualized under the fluorescence microscope (Olympus, Japan).

### Transwell assays

The procedures for transwell migration and invasion experiments were performed as previously described 17, 18. Transwell inserts (8.0 µm pore size, Corning, USA) with and without matrigel-precoated were seeded with 10^5^ PTC cells. DMEM medium supplementing 20% FBS was utilized for chemoattractant in the lower wells. After incubating for 24 hours (migration) or 48 hours (invasion), the upper cells were fixed by 4% paraformaldehyde and stained with 0.3% crystal violet. For wound-healing experiments, PTC cells were cultured in 6-well plates at 100% confluence and scratched with sterile micropipette tip, and then the cells were washed with PBS and cultured in serum-free medium. Images were taken at the indicated time under the microscope.

### Enzyme-linked immunosorbent assay (ELISA)

Human IL-6 Quantikine ELISA Kit was purchased from R&D systems (Minneapolis, USA) and performed according to the manufacturer's instructions. Culture supernatants from BCPAP and KTC-1 cells with indicated vectors were collected and added to the ELISA plates previously coated with the capture antibody. Next, the wells were washed with PBS and incubated with detection antibody. IL-6 protein concentration was determined basing on the standard curve.

### Immunohistochemistry

Paraffin-embedded PTC samples and tumor tissues from xenograft models were sliced into 4.0 μm thickness. After deparaffinization, hydration, and antigen retrieval, tissue slides were incubated with anti-ANXA1 (#32934, Cell Signaling Technology) or anti-Ki67 (ab15580, Abcam) at 4 °C overnight. The ANXA1 immunoreactivity score was calculated by two independent researchers based on the area of positive staining-tumor cells and the intensity of staining.

### Animal models

All animal procedures were approved by the Animal Ethics Committee of the First Affiliated Hospital of Zhengzhou University. Four to six-week-old female BALB/c nude mice were purchased from the Shanghai SLAC Laboratory Animal. 5 × 10^6^ either scramble BCPAP or shANXA1 BCPAP cells in 150 µL PBS were injected hypodermically in the right armpits of nude mice (five mice per group). Tumor volumes were measured every five days using digital calipers by the same researcher to avoid random errors, and calculated using established formula (length × width^2^)/2. Thirty days after injection, mice were euthanized and tumors were harvested and fixed in 4% paraformaldehyde for further analyses. For* in vivo* metastatic model, 2 × 10^6^ BCPAP control and shANXA1 cells were administrated to the tail-veins of nude mice. After 8 weeks, the lungs were examined and metastatic lesions were quantified and collected for histological determination.

### Statistical analysis

All results are presented as the mean ± standard deviation (SD) from at least three independent experiments and statistical analyses were undertaken by GraphPad 7.0 Software. Two-tailed Student's t-test was employed for comparisons between two individual groups and the Chi-square test was used for testing the categorical variables. Pearson correlation was used to evaluate the association between ANXA1 and IL-6. P < 0.05 was considered statistically significant.

## Results

### Abundant ANXA1 predicts poor clinical outcomes in PTC

**Figure 1:**
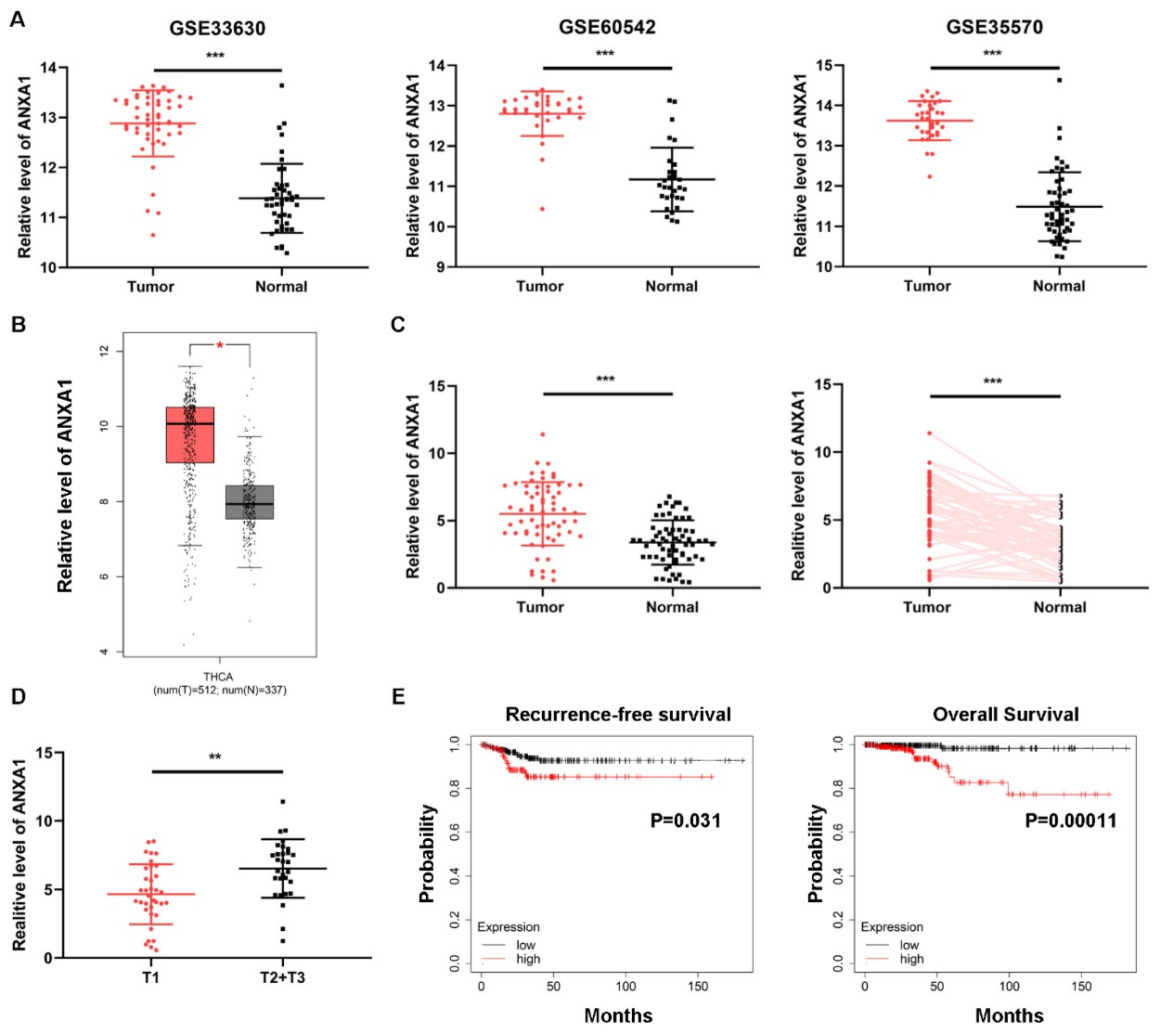
** Overexpression of ANXA1 is correlated with primary tumor size in PTC. (A)** GEO datasets results revealed that ANXA1 was upregulated in PTC tumor tissues than normal thyroid tissues.** (B)** Expression profiles of ANXA1 in PTC patients from the GEPIA platform of TCGA data.** (C)** The mRNA level of ANXA1 in paired PTC patients' samples (n=64). **(D)** Relative expression of ANXA1 in PTC patients with different tumor sizes (T1 vs. T2+T3).** (E)** Kaplan-Meier analysis showed that high ANXA1 expression predicted poor overall survival and recurrence-free survival of PTC patients. **P < 0.01, ***P < 0.001.

Intending to clarify the specific role of ANXA1 in PTC carcinogenesis, we first investigated the expression of ANXA1 using data from public databases. Three GEO datasets containing over one hundred PTC samples and normal thyroid tissues, all confirmed that ANXA1 was remarkably upregulated in PTC tissues (Fig. 1A). In addition, TCGA THCA (thyroid carcinoma) data extracted from GEPIA website also showed that ANXA1 was increased in PTC samples (Fig. 1B). We further analyzed the expression profiles of ANXA1 in our cohort consisting of 64 PTC patients, RT-PCR results demonstrated that ANXA1 was prominently upregulated in PTC specimens compared to matched adjacent non-tumor tissues (Fig. 1C). Notably, we found that ANXA1 expression was positively correlated with the tumor volume of PTC patients (Fig. 1D). Patients with larger tumor size often exhibited the elevated level of ANXA1 in comparison with those with smaller tumor size (Table 1). Besides, we evaluated the role of ANXA in predicting the survival of PTC patients using data from Kaplan-Meier Plotter. High ANXA1 expression was associated with a significantly decreased recurrence-free survival and overall survival, suggesting ANXA1 may serve as a prognostic marker in PTC (Fig. 1E).

**Figure 2:**
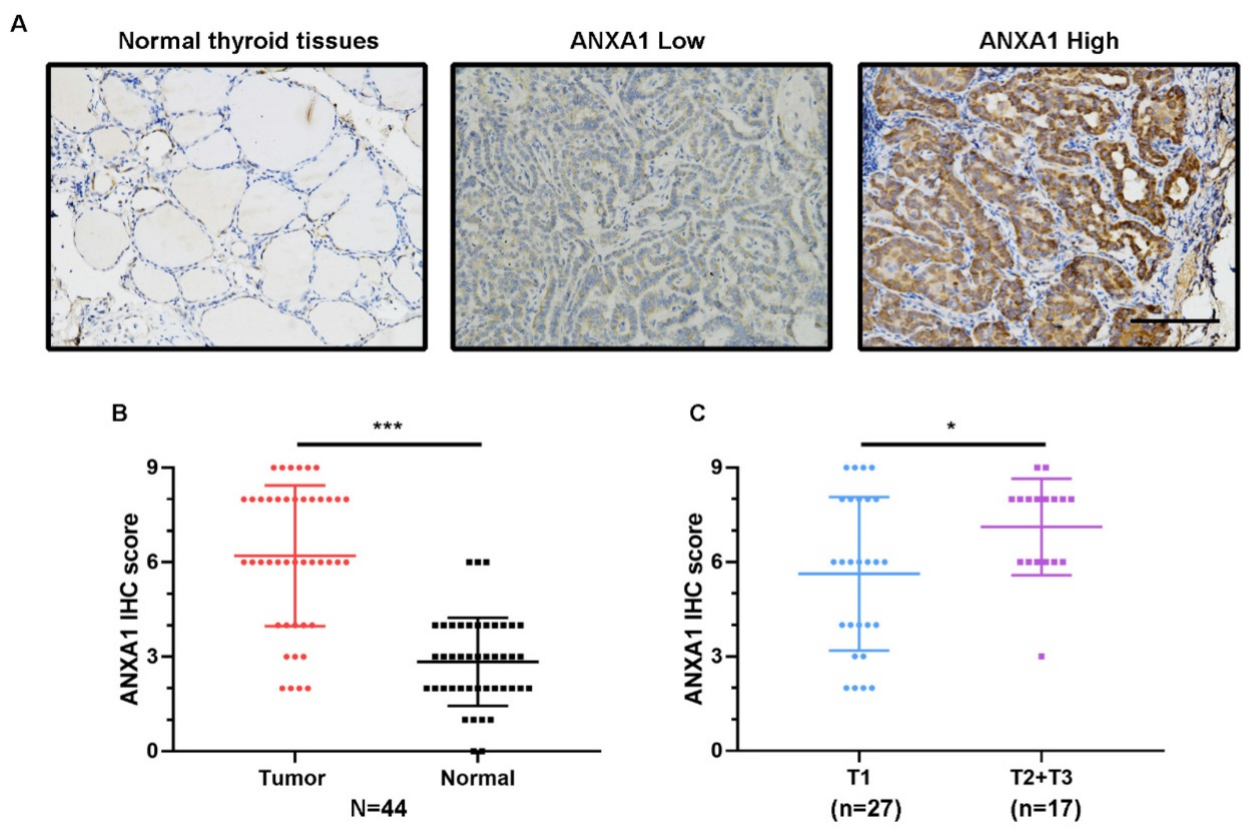
** ANXA1 protein level is elevated in PTC tissues. (A)** Representative immunohistochemistry images of ANXA1 expression in normal thyroid tissues and PTC tissues. **(B)** Analysis of ANXA1 protein level based on immunohistochemistry scores from 44 PTC patients. **(C)** T2 and T3 PTC patients showed higher ANXA1 protein expression compared to T1 patients. Scale bar: 200 μm. *P < 0.05, ***P < 0.001.

Next, we conducted immunohistochemistry staining for ANXA1 using 44 PTC patient specimens. Corroborating the elevated mRNA levels, ANXA1 exhibited the intense staining in most of the PTC tissues slides, while the matching normal thyroid tissues staining was very weak and barely visible (Fig. 2A). We measured the immunohistochemical score for each PTC patient and the results showed that the average scores of ANXA1 staining were much higher in tumor areas than neighboring normal thyroid regions (Fig. 2B). Meanwhile, compared with PTC patients with smaller tumor size (no more than two centimeters), PTC patients in T2 and T3 status showed higher ANXA1 protein levels (Fig. 2C). Taken together, our results revealed that ANXA1 was profoundly upregulated in PTC tissues, whose expression is closely related to tumor size and adverse prognosis in PTC patients.

### Overexpression of ANXA1 accelerates PTC cell proliferation

**Figure 3:**
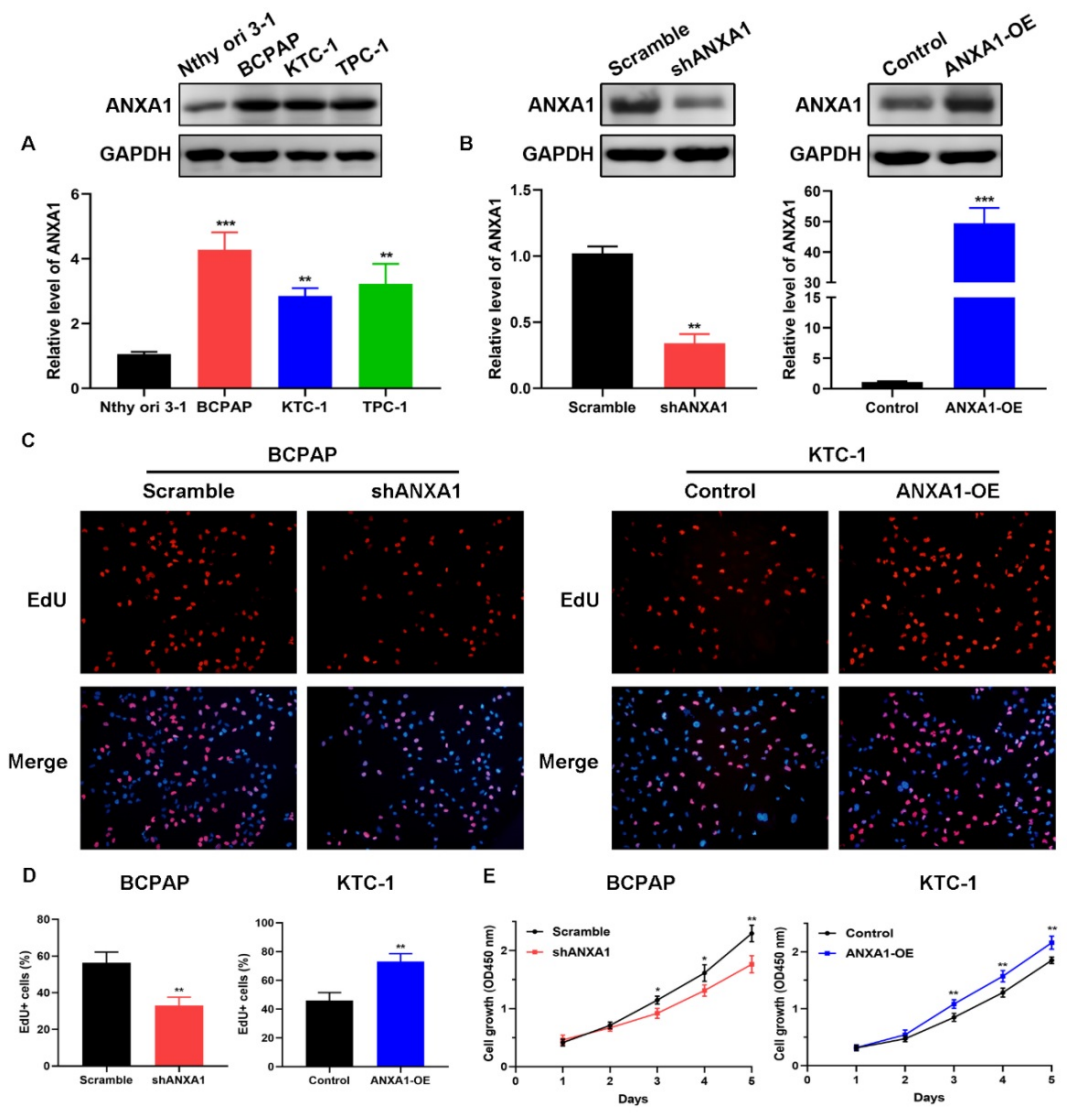
** ANXA1 accelerates PTC cell growth *in vitro*. (A)** ANXA1 was widely increased in PTC cell lines compared to thyroid epithelial cells. **(B)** RT-PCR and western blot analysis confirmed the successful construction of ANXA1 knock-down and overexpression plasmids in BCPAP and KTC-1 cells, respectively. **(C)** DNA synthesis was examined in BCPAP cells stably expressing shANXA1 vector and KTC-1 cells stably expressing ANXA1 and their control cells using EdU assays.** (D)** Quantitative analysis of EdU positive BCPAP and KTC-1 cells was presented as mean ± SD. **(E)** Cell viability of PTC cells upon inhibition or overexpression of ANXA1 was monitored by CCK-8 assays. *P < 0.05, **P < 0.01, ***P < 0.001.

To explore the biological functions of ANXA1 in PTC, we detected the expression of ANXA1 among a panel of PTC cell lines. BCPAP cells exhibited the most abundant endogenous expression of ANXA1 when compared with the immortalized normal thyroid epithelial cell line Nthy-ori 3-1, while KTC-1 cells harbored the lowest ANXA1 mRNA level, as indicated by RT-PCR and western blot (Fig. 3A). Therefore, we utilized these two cells for functional studies. shRNA-mediated knockdown was applied to BCPAP cells and ANXA1 overexpression vector was transfected into KTC-1 cells. ANXA1 was efficiently silenced in BCPAP cells and overexpressed in KTC-1 cells, as manifested by RT-PCR analysis and immunoblotting (Fig. 3B). We proceeded to perform the EdU incorporation assays to assess the effects of ANXA1 on PTC cell viability. We found that ANXA1 deficiency substantially suppressed the proliferation of BCPAP cells. On the contrary, ectopic expression of ANXA1 increased the number of EdU positive KTC-1 cells (Fig. 3C and D). Additionally, CCK-8 experiments indicated that inhibition of ANXA1 apparently diminished cell proliferation, while overexpression of ANXA1 accelerated PTC cell growth *in vitro* (Fig. 3E). Collectively, the above results demonstrated that ANXA1 expedites the growth of PTC cells.

### ANXA1 contributes to migration and invasion of PTC cells through epithelial-mesenchymal transition

**Figure 4:**
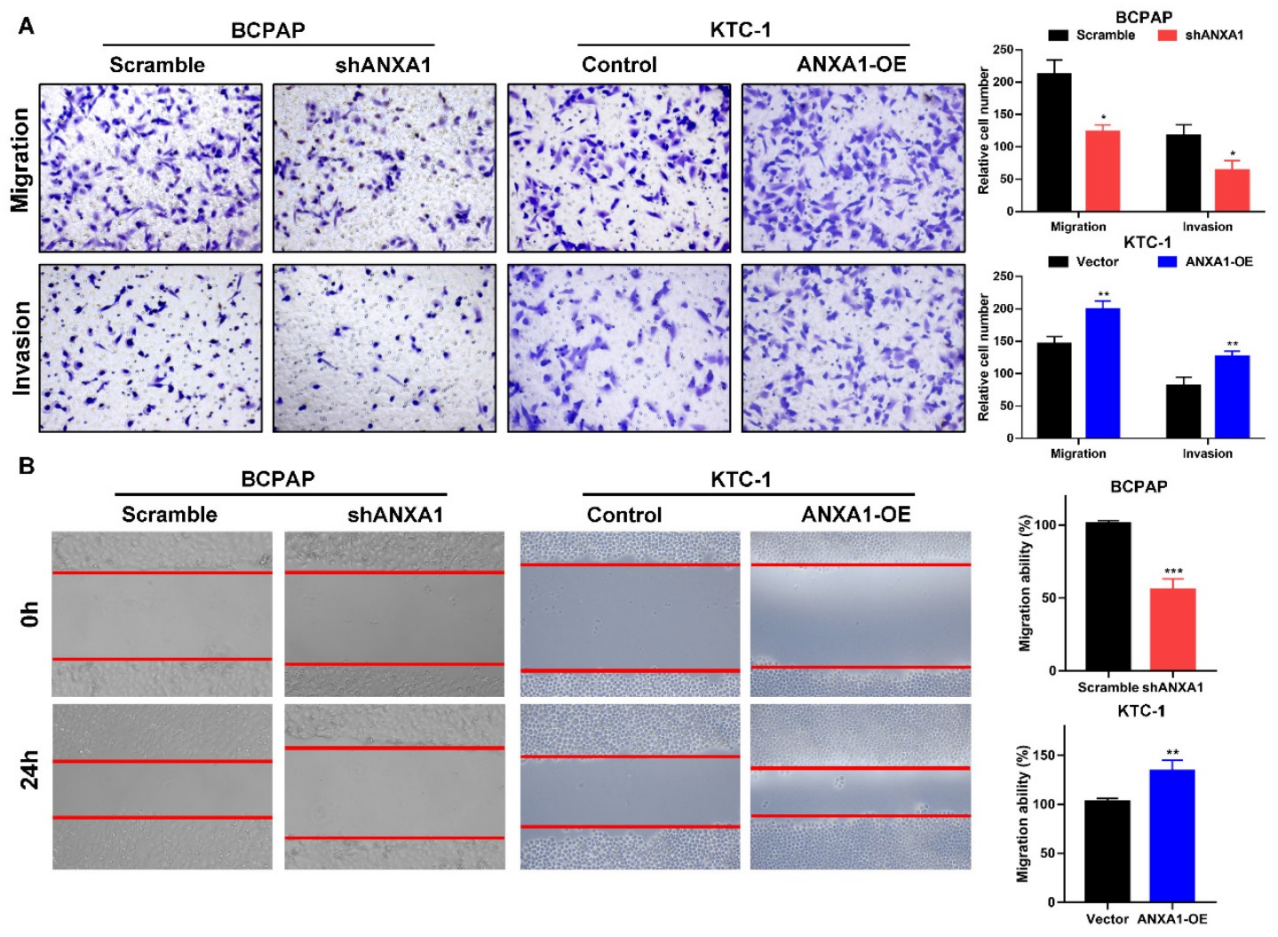
**ANXA1 promotes cell migration and invasion of PTC cells. (A)** The effects of ANXA1 overexpression and knock-down on PTC cell migration and invasion were investigated by transwell experiments.** (B)** Wound-healing assays showed that ANXA1 regulated PTC cell motility. Quantification of wound closure was presented. Magnification: × 100. *P < 0.05, **P < 0.01, ***P < 0.001.

**Figure 5:**
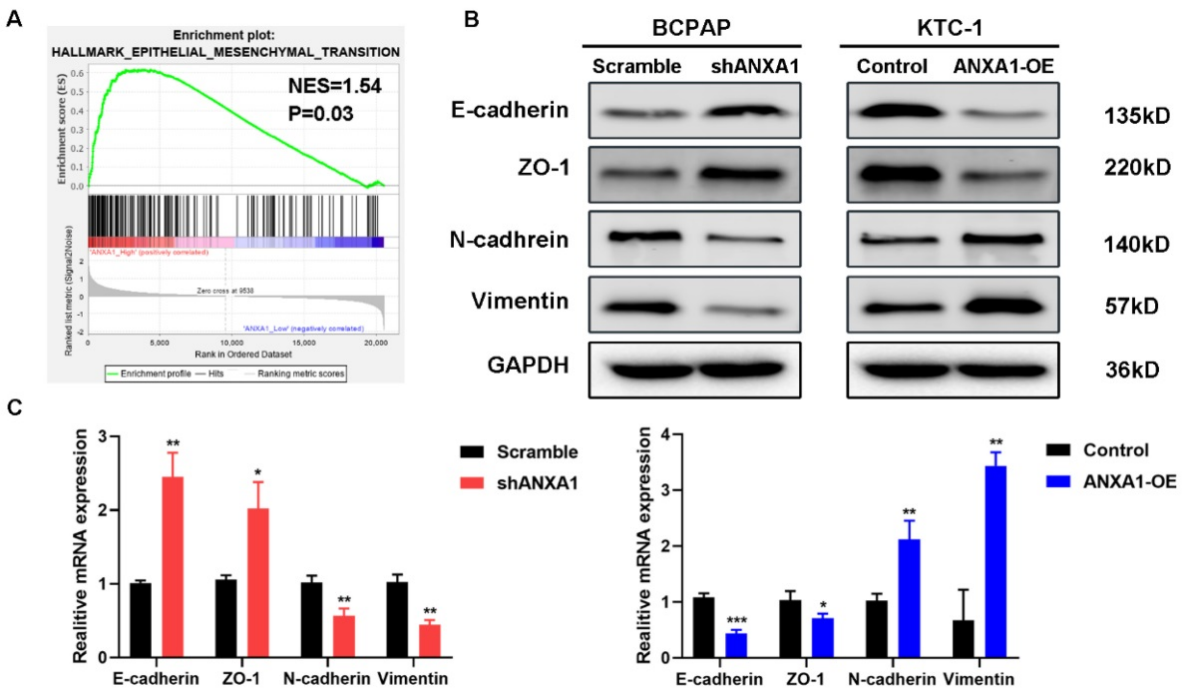
**ANXA1 regulates cell migration through epithelial-mesenchymal transition. (A)** GSEA analysis demonstrated a positive correlation between ANXA1 enrichment and epithelial-mesenchymal transition in PTC. NES: normalized enrichment score.** (B)** Western blot analysis showing the expression of E-cadherin, ZO-1, N-cadherin and Vimentin was changed after inhibition or overexpression of ANXA1 in PTC cells. GAPDH was used as a loading control.** (C)** The mRNA expression of EMT-related genes was detected by RT-PCR from three independent experiments. *P < 0.05, **P < 0.01, ***P < 0.001.

Next, we carried out transwell assays to determine the influence of ANXA1 on the metastasis of PTC cells. Strikingly, ANXA1 knockdown dramatically inhibited BCPAP cell migration and invasion. Not surprisingly, overexpression of ANXA1 showed the opposite effects on PTC cell mobility (Fig. 4A). We further confirmed these results by wound-healing assays. Consistent with the results from transwell experiments, ANXA1 significantly drove PTC cell migration *in vitro* (Fig. 4B). We also established ANXA1 overexpression vectors in BCPAP cells, and the data showed that enforced ANXA1 expression aggravated the growth and migration of BCPAP cells, strengthening the accuracy of our findings (Supplementary Figure 1). To delineate the molecular mechanism through which ANXA1 participated in PTC metastasis, RNA-sequencing profiles from the TCGA THCA database were analyzed. We divided PTC patients into two groups according to the expression level of ANXA1. Gene set enrichment analysis (GSEA) was performed to identify the differentially expressed pathways between these two groups. Interestingly, we found that epithelial-mesenchymal transition, a crucial biological process during tumor metastasis, was enriched in PTC samples with ANXA1 high expression (Fig. 5A). We further detected the levels of EMT markers by western blotting, including the epithelial marker E-cadherin and ZO-1, as well as the mesenchymal markers N-cadherin and Vimentin. Knockdown of ANXA1 reduced the protein levels of N-cadherin and Vimentin while induced an increase of E-cadherin and ZO-1. In contrast, overexpression of ANXA1 promoted the expression of N-cadherin and Vimentin and reduced the level of E-cadherin and ZO-1 (Fig. 5B). RT-PCR results also confirmed the EMT-related makers were modulated by ANXA1 in PTC cells (Fig. 5C). These findings suggested that ANXA1 may deregulate EMT thereby affecting PTC cells migration and invasion.

### ANXA1 overexpression activates the IL-6/JAK2/STAT3 pathway in PTC

**Figure 6:**
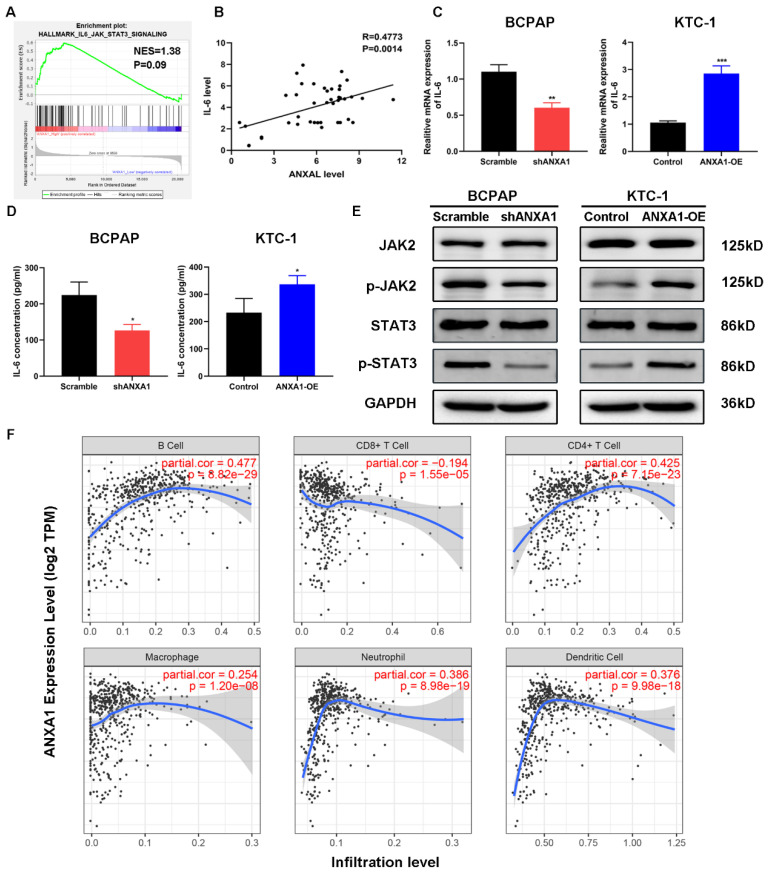
** ANXA1 activates IL-6/JAK2/STAT3 pathway in PTC cells. (A)** GSEA enrichment analysis identified that IL-6/JAK2/STAT3 pathway was associated with SLC34A2 high expression.** (B)** The relationship between ANXA1 and IL-6 mRNA expression was analyzed in 42 PTC patients.** (C)** RT-PCR showed that IL-6 expression was regulated by ANXA1 in PTC cells.** (D)** ELISA analysis of IL-6 concentrations from the supernatants of indicated PTC cells. **(E)** Protein levels of JAK2, p-JAK2, STAT3 and p-STAT3 were determined by immunoblotting in PTC cells stably expressing shANXA1 and ANXA1-OE vectors.** (F)** Correlation between ANXA1 expression and infiltration levels of different immune cells in PTC. *P < 0.05, **P < 0.01, ***P < 0.001.

Among the enrichment pathways or biological processes, we noticed that IL-6/JAK2/STAT3 pathway was positively related to the expression of ANXA1 in PTC (Fig. 6A). To this end, we firstly detected the relationship between ANXA1 and IL-6 in forty-two PTC samples. RT-PCR analysis demonstrated that that expression of ANXA1 and IL-6 was strongly correlated (R=0.4773, P=0.0014, Fig. 6B). We next tested whether ANXA1 could regulate IL-6 expression. As depicted in Fig. 6C, ectopic expression of ANXA1 increased the level of IL-6 while ANXA1 deficiency decreased the expression of IL-6. Moreover, we examined the IL-6 concentrations from the supernatants of PTC cells upon overexpression and depletion of ANXA1. ANXA1 promoted the IL-6 production in PTC cells and vice versa, as evidenced by ELISA experiments (Fig. 6D). We further analyzed the change of IL-6 downstream markers by western blot. Overexpression of ANXA1 substantially augmented the phosphorylation of JAK2 and STAT3 without altering total JAK2 and STAT3 protein level, whereas ANXA1 knockdown showed the opposing trends (Fig. 6E). IL-6/STAT3 signaling pathway has been found to play a vital role in creating the inflammatory microenvironment during carcinogenesis, which prompted us to explore the correlation between ANXA1 and immune cells infiltration in PTC. By analyzing the data from Tumor Immune Estimation Resource (TIMER), we found that ANXA1 expression closely correlated with the infiltration level of B cell (R=0.477), CD4+ T cell (R=0.425), neutrophil (R=0.386) and dendritic cell (R=0.376) in PTC, implying ANXA1 may influence the chemotaxis of certain types of immune cells to mediate the immune escape in the PTC microenvironment (Fig. 6F).

### ANXA1 depletion impedes tumor formation and metastasis *in vivo*

**Figure 7:**
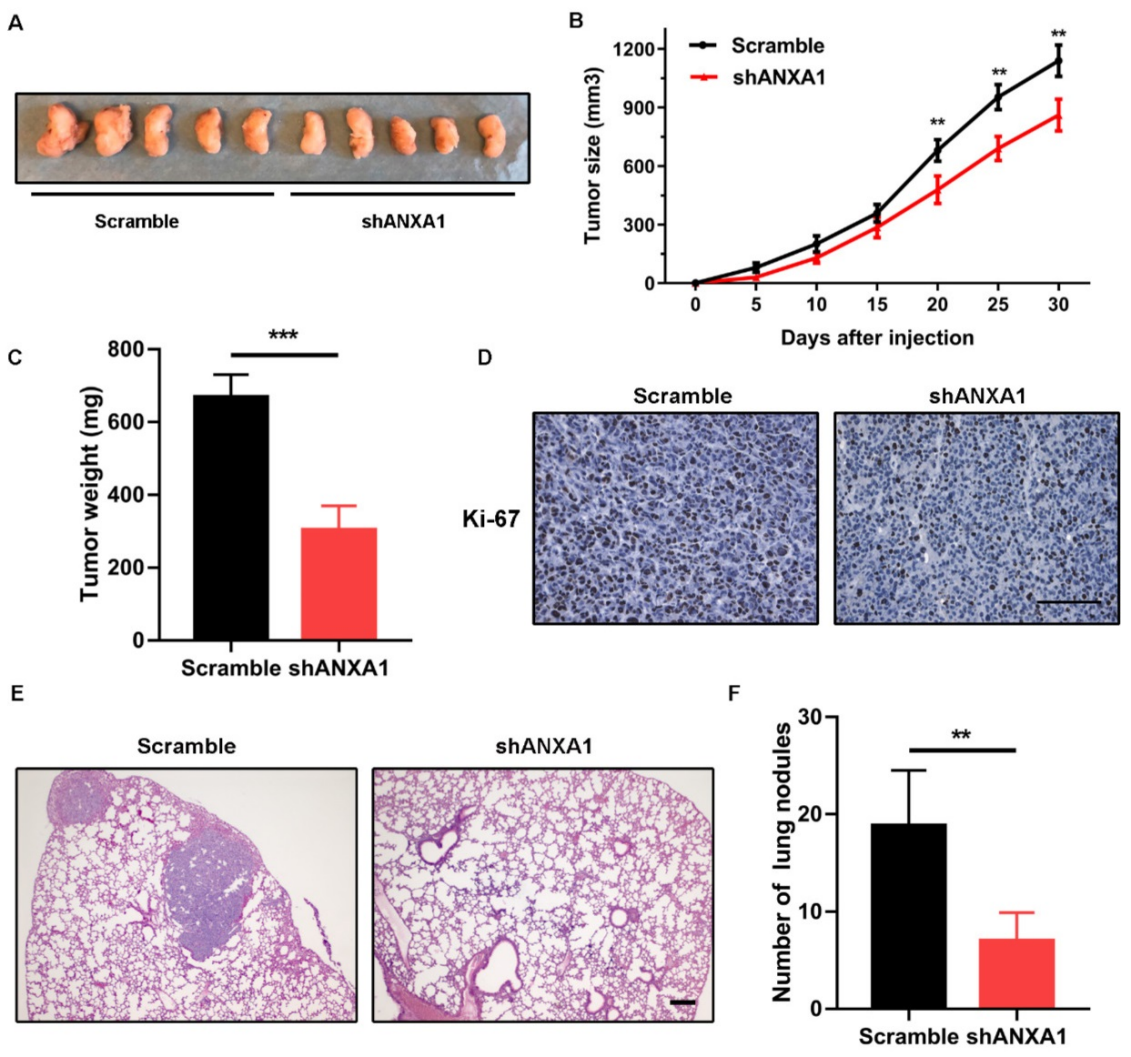
** Silencing of ANXA1 retards tumor growth of PTC cells *in vivo*. (A)** Representative images of tumors from xenograft models after injection of control and ANXA1-knockdown cells (five mice per group). **(B)** Relative tumor growth curve was obtained from two groups of mice, monitoring every five days.** (C)** Tumor weights were quantified from indicated mice.** (D)** Immunohistochemical staining results demonstrating that Ki-67 level was decreased accompanied by inhibition of ANXA1 in tumor sections. Scale bar: 200 μm. **(E)** Representative H&E staining images of lungs nodules were presented from control and ANXA1-knockdown cells. Scale bar: 200 μm.** (F)** Number of metastatic foci was quantified from indicated mice (five mice per group). **P < 0.01, ***P < 0.001.

To confirm the accuracy of *in vitro* results, we performed subcutaneous injection of the scramble control and shANXA1 BCPAP cells to Balb/c nude mice. Since 20 days after tumor cell implantation, we noticed that tumors from the control group were obviously larger than those from control mice. At the observing endpoint, tumor tissues were excised from both groups and quantified for average tumor weights. The *in vivo* data displayed that silencing of ANXA1 retarded the tumor growth of PTC cells (Fig. 7A-C). Meanwhile, we conducted the immunohistochemistry staining for tissue slides from these two groups. ANXA1-silenced tumor cells showed a dramatic reduction of Ki-67 staining compared to control cells, indicating *in vivo* tumor cell proliferation was robustly hindered by silencing of ANXA1 (Fig. 7D). In addition, we determined the effects of ANXA1 on PTC cell *in vivo* metastasis by tail-vein injection. Eight weeks after injection, we observed that mice from shANXA1 group possessed fewer lung metastatic nodules than control group, suggesting ANXA1 regulated *in vivo* metastasis of PTC cells. (Fig. 7E and F).

## Discussion

Accumulating evidence denoted that aberrant expression of ANXA1 may represent potential diagnostic and treatment biomarkers in numerous human diseases, including cancer. Giusti et al. previously performed mass spectrometry proteome analysis using thyroid samples collected from fine-needle aspiration (FNA) biopsy to define the benign or malignant status of these thyroid lesions. They found seventeen proteins were upregulated in classical variant PTC (cPTC) and tall cell variant PTC (TcPTC) with respect to benign thyroid nodules and ANXA1 was one of them 19. Subsequently, this research group used pre-surgical FNA specimens from benign thyroid nodules, cPTCs and TcPTCs to measure the applicability of identified proteins in distinguishing different functional properties of thyroid nodules. Receiver operating characteristic (ROC) curve showed that ANXA1 was better than other proteins and possessed a sensitivity of 87 and 84.6% and a specificity of 94.3 and 100% for cPTC and TcPTC respectively, indicating ANXA1 was an ideal marker to separate malignant and benign thyroid lesions 20. In another study, they also found that combining a panel of proteins including ANXA1, ENO1, DJ-1, SOD, and CRNN for preoperative diagnosis of thyroid cancer to avoid unnecessary thyroidectomy 21. Consistent with previous studies, we confirmed the increased level of ANXA1 in PTC tissues in our cohorts. More importantly, we discovered the high level of ANXA1 was closely linked to the tumor size of PTC samples and predicted unfavorable prognosis of patients, emphasizing the important clinical significance of ANXA1 in PTC patients.

Metastatic spread of tumor cells is responsible for most of the tumor patients' mortality 22, 23. It is well known that EMT is an essential biological process to allow successful formation of metastasis to distant organs, which is characterized by adherent epithelial cells acquire the ability to change to mesenchymal cell phenotype and disseminate into blood circulation. On the contrary, mesenchymal-to-epithelial transition (MET) help tumor cells to restore the epithelial properties and form metastatic foci at distant sites 24. Previous studies indicated EMT enhanced the migratory capacity, invasiveness, and evoked resistance to apoptosis in PTC cells 25, 26. To understand the mechanisms involved in ANXA1-induced PTC cell migration and invasion, the effects of ANXA1 on EMT-related markers were examined. Our study illustrated that forced ANXA1 expression rendered the expression of extracellular matrix constituents such as N-cadherin and Vimentin as well as decreased epithelial hallmarks E-cadherin and ZO-1 at both mRNA and protein levels. Maschler et al. reported that ANXA1 functioned as a potent suppressor of EMT in breast cancer. Knockdown of ANXA1 stimulated tumor growth and induced EMT in murine mammary epithelial cell line 27. Another report showed that ANXA1-suppressed autophagy induced EMT-like changes of the nasopharyngeal carcinoma cells and promoted *in vivo* lung metastases 28. These phenotypic differences may be attributed to the genetic heterogeneity in different tumor types. As a class of Ca^2+^-regulated proteins, annexins possess the unique structure of their conserved Ca^2+^ binding regions, which links them to a series of membrane-trafficking events, including membrane-cytoskeleton linkages, Ca^2+^-evoked exocytosis and endocytosis and the ion fluxes across membranes 29. These crucial effects of annexin family members on membrane organization and traffic may explain why the dysregulation of ANXA1 influences the epithelial-mesenchymal plasticity in cells.

Scientists have been investigating the mechanisms underlying the JAK2/STAT3 pathway mediated tumorigenesis for years. IL-6 is known as the classic activator of the JAK2/STAT3 cascade. Once JAK2 protein was activated by proinflammatory cytokine IL-6, STAT3 can be phosphorylated at Tyr-705, formed dimerization, and translocated to the cell nucleus, in which p-STAT3 functions as transcription factor relying on its DNA binding domain to regulate downstream genes expression 30. STAT3 signaling is critical for driving tumor growth, migration, angiogenesis, and inflammatory cross-talk with immune cells during the carcinogenesis 31, 32. However, the modulator of the IL-6/STAT3 pathway during the development of PTC is still obscure. In this study, we observed the IL-6 level was positively correlated with ANXA1 in PTC samples and the secretion of IL-6 into the PTC cells supernatant was increased upon ANXA1 overexpression. ANXA1 also elicited the phosphorylation of JAK2 and STAT3 to activate downstream oncogenes. Besides, we found that ANXA1 is positively correlated with certain types of immune cell infiltration, including B cell, CD4+ T cell, neutrophil and dendritic cell, suggesting ANXA1 may activate the IL-6/STAT3 pathway to recruit these immune cells to form an immune suppression microenvironment to promote PTC development. However, the precise mechanisms concerning ANXA1-mediated immune cells infiltration warrant further investigation.

Herein, we provide the first evidence that ANXA1 is a positive regulator of tumor growth and metastasis in PTC, complementing its biological function via influencing the dynamic properties of EMT and regulating IL-6/JAK2/STAT3 signaling network. Moreover, inhibition of ANXA1 showed the profound effects in retarding PTC cell growth and metastasis in mouse models. Overall, our findings define a valid prognostic biomarker and useful treatment target for combating PTC growth and metastasis.

## Supplementary Material

Supplementary figure S1.

## Figures and Tables

**Table 1:** Correlation between mRNA expression of ANXA1 and clinical characteristics in 64 PTC patients.

Characteristics	No. of patients	ANXA1 expression		*P*-value
		High group	Low group	
Gender				
Male	24	14(43.75%)	10(31.25%)	0.3017
Female	40	18(56.25%)	22(68.75%)	
Age				
<45	31	15(46.88%)	16(50%)	0.8025
≥45	33	17(53.12%)	16(50%)	
T stage				
T1	35	12(37.50%)	23(71.88%)	0.0057**
T2+T3	29	20(62.50%)	9(28.12%)	
N stage				
N0	30	13(40.63%)	17(53.12%)	0.3164
N1a+N1b	34	19(59.37%)	15(46.88%)	
TNM stage				
I+II	39	18(56.25%)	21(65.63%)	0.4421
III+IV	25	14(43.75%)	11(34.37%)	
Unilateral or Bilateral Unilateral Bilateral Multifocality	39 25	17(53.13%) 15(46.87%)	22(68.75%) 10(31.25%)	0.2002
Present	21	11(34.38%)	10(31.25%)	0.7901
Absent	43	21(65.62%)	22(68.75%)	

**P < 0.01
