# Supplementary material for: ANXA1 enhances tumor proliferation and migration by regulating epithelial-mesenchymal transition and IL-6/JAK2/STAT3 pathway in papillary thyroid carcinoma
Source: J Cancer. 2021 Jan 1;12(5):1295–306. doi: 10.7150/jca.52171 (PMC7847635; doi:10.7150/jca.52171)
Supplement: Supplementary file 1 — Supplementary figure S1. [file jcav12p1295s1.pdf]

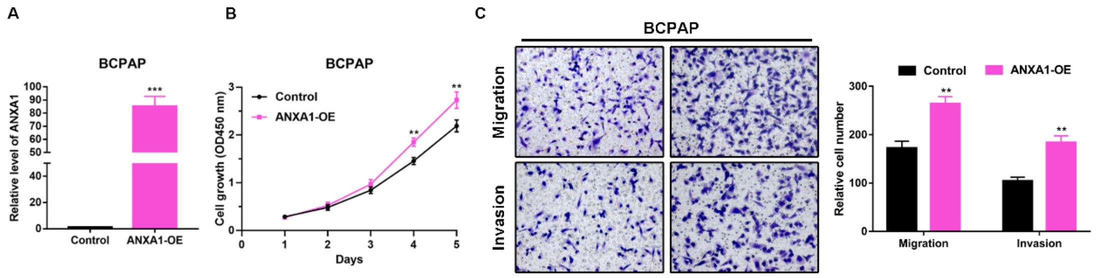

Supplementary Figure 1. Overexpression of ANXA1 promotes PTC cells growth and migration *in vivo*. (A) RT-PCR analysis of ANXA1 expression in BCPAP transfected with ANXA1 plasmid and empty vector. **(B)** Cell proliferation was examined by CCK-8 in BCPAP cells. **(C)** Migration and invasion capacities of BCPAP cells were evaluated with transwell assays after overexpression of ANXA1.
